# Atypical multisensory integration in Niemann-Pick type C disease – towards potential biomarkers

**DOI:** 10.1186/s13023-014-0149-x

**Published:** 2014-09-20

**Authors:** Gizely N Andrade, Sophie Molholm, John S Butler, Alice B Brandwein, Steven U Walkley, John J Foxe

**Affiliations:** Department of Pediatrics, The Sheryl and Daniel R. Tishman Cognitive Neurophysiology Laboratory, Children’s Evaluation and Rehabilitation Center (CERC), Albert Einstein College of Medicine & Montefiore Medical Center, Van Etten Building – Wing 1C, 1225 Morris Park Avenue, Bronx, NY 10461 USA; Program in Cognitive Neuroscience, The Graduate Center of the City University of New York, 365 Fifth Avenue, New York, NY 10016 USA; Trinity Centre for Bioengineering, Trinity College, Dublin 2, Ireland; Trinity College Institute of Neuroscience, Trinity College, Dublin 2, Ireland; The Dominick P. Purpura Department of Neuroscience, Rose F. Kennedy Intellectual and Developmental Disabilities Research Center, Albert Einstein College of Medicine, Bronx, NY 10461 USA

**Keywords:** Race model, Neurodegeneration, NPC1, NPC2, Lysosomal disease, Cross-modal, Rare disease, Sensory processing, Audio-visual, Sensory integration

## Abstract

**Background:**

Niemann-Pick type C (NPC) is an autosomal recessive disease in which cholesterol and glycosphingolipids accumulate in lysosomes due to aberrant cell-transport mechanisms. It is characterized by progressive and ultimately terminal neurological disease, but both pre-clinical studies and direct human trials are underway to test the safety and efficacy of cholesterol clearing compounds, with good success already observed in animal models. Key to assessing the effectiveness of interventions in patients, however, is the development of objective neurobiological outcome measures. Multisensory integration mechanisms present as an excellent candidate since they necessarily rely on the fidelity of long-range neural connections between the respective sensory cortices (e.g. the auditory and visual systems).

**Methods:**

A simple way to test integrity of the multisensory system is to ask whether individuals respond faster to the occurrence of a bisensory event than they do to the occurrence of either of the unisensory constituents alone. Here, we presented simple auditory, visual, and audio-visual stimuli in random sequence. Participants responded as fast as possible with a button push. One 11-year-old and two 14-year-old boys with NPC participated in the experiment and their results were compared to those of 35 age-matched neurotypical boys.

**Results:**

Reaction times (RTs) to the stimuli when presented simultaneously were significantly faster than when they were presented alone in the neurotypical children, a facilitation that could not be accounted for by probability summation, as evidenced by violation of the so-called ‘race’ model. In stark contrast, the NPC boys showed no such speeding, despite the fact that their unisensory RTs fell within the distribution of RTs observed in the neurotypicals.

**Conclusions:**

These results uncover a previously undescribed deficit in multisensory integrative abilities in NPC, with implications for ongoing treatment of the clinical symptoms of these children. They also suggest that multisensory processes may represent a good candidate biomarker against which to test the efficacy of therapeutic interventions.

**Electronic supplementary material:**

The online version of this article (doi:10.1186/s13023-014-0149-x) contains supplementary material, which is available to authorized users.

## Background

Niemann-Pick type C (NPC) disease is a rare progressive lysosomal storage disorder caused by mutations in either the *NPC1* or *NPC2* gene, with about 95% of cases attributable to the former [1,2]. Individuals with NPC cannot properly metabolize cholesterol and other lipids which accumulate in the brain and in visceral organs (e.g. liver and spleen), ultimately causing cell dysfunction and organ system failure. Although NPC1 and NPC2 proteins are expressed ubiquitously, brain tissue is the most severely affected, resulting in widespread intraneuronal storage of cholesterol and glycosphingolipids that ultimately results in massive neurodegeneration [3–6]. While appearing relatively typical during the early stages of the disease, over time NPC children develop vertical gaze palsy, motor system impairment, learning difficulties and clumsiness, as well as seizures [7–9]. Documented changes in brain include ectopic dendrite growth, altered synaptic connectivity affecting cortical pyramidal neurons, axonal degeneration, myelin loss, gliosis and the formation of neurofibrillary tangles similar to Alzheimer's disease [10,11]. Neuronal death is prominent in some brain regions such as the cerebellum where Purkinje cells selectively die, undoubtedly contributing to the clinically-evident motor system dysfunction [5,10,12]. Effective treatments are limited, although promising clinical trials are underway based on results in animal models of NPC [11,13,14].

Key to advancing new treatments for this and related lysosomal diseases with neural involvement is the development of objective biomarkers of neurological function against which the efficacy of new drugs can be tested in human patients. Our work and that of others has demonstrated the essential role that multisensory integration (MSI) plays in typical perception and cognition [15–24]. Because inputs from the various senses (e.g., the auditory, visual and somatosensory systems) initially arrive into widely separated regions of the neocortex, MSI must involve ongoing communication between relatively far-flung cortical regions, although it may well be initiated even earlier in the hierarchy within nuclei of the thalamus [25]. In this sense, probing multisensory functioning provides an excellent assay of inter-regional communication, and the fidelity of the multisensory system must at least in part be a function of the integrity of long-range neural connectivity. For this reason we expected measures of MSI to provide a sensitive metric of neural dysfunction in NPC disease. What's more, MSI processes show a prolonged period of neuroplasticity, with continued development of these abilities seen into the late teenage years [22,26]. As such, measures of MSI may provide useful biomarkers against which to test the impact of treatment on brain function.

A straightforward way to measure multisensory integration is to compare reaction times (RT) to unisensory and multisensory events during a simple speeded response task. It has been firmly established that adults react more quickly to multisensory than unisensory inputs [21,27–30]. For such behavioral facilitation to be unequivocally attributed to *multisensory integration*, this speeding up must exceed what is predicted due to the mere presence of a redundant signal (i.e. two inputs). That is, when two stimulus copies are presented simultaneously, even if both were to be processed entirely independently in the brain, one would still expect to see a speeding up of responses since there is increased likelihood that either of the two stimuli will yield a fast reaction-time relative to just one input. This is often referred to as the *Redundant Signals Effect (RSE),* and its presence does not, of itself, necessarily point to integration effects. The so-called “race model” is applied to test for the presence of true multisensory effects, by assessing whether responses to multisensory inputs are faster than the fastest possible responses produced by the unisensory conditions [31–33]. This is achieved by comparing the probabilities of making fast responses during multisensory events to those during unisensory events. The race model is said to be violated whenever the cumulative probability (CP) of a response at a given latency for the multisensory condition is greater than the sum of the CPs from each of the unisensory conditions. When the race model is violated, it is taken to be a strong indication that the inputs from the two different senses are interacting (in a non-additive way) to produce the speeding of the responses. Work from our laboratory suggests that this metric of MSI RT-speeding follows a developmental trajectory, with little evidence for behavioral enhancement before age 9, but that near full maturity is reached by age 16 [26,34]. Moreover, in these developmental studies, behavioral performance was shown to benefit from MSI at the single participant level for 95% of neurotypical participants aged 11-16, and 100% of participants aged 13-16. This relatively protracted developmental trajectory of MSI behavioral facilitation is consistently seen across laboratories [35,36]. Here we used this behavioral approach to assay multisensory function in three boys with NPC -- two adolescents (14 years, 7 months & 14 years, 5 months old) and one younger boy (11 years, 1 month) -- comparing their performance to that of 16 neurotypical adolescent boys aged 13-15, and 19 neurotypical boys aged 10-13, respectively.

## Methods

### Participants

Two adolescent boys with NPC (14 years, 7 months & 14 years, 5 months of age respectively) and one 11 year old boy with NPC (11 years, 1 month) participated in the study. NPC was clinically diagnosed by metabolic specialists and confirmed via genetic testing. Participants were administered the Wechsler Abbreviated Scales of Intelligence (WASI-II) The WASI-II is a short and reliable measure of intelligence that assesses general intellectual functioning. All four subtests were used: *Vocabulary, Block Design, Similarities*, and *Matrix Reasoning. Vocabulary* measures the individual’s expressive vocabulary, verbal knowledge, and fund of information. *Block Design* measures spatial visualization, visual-motor coordination, and abstract conceptualization. The *Similarities* subtest measures verbal concept formation, abstract verbal reasoning ability, and general intellectual ability. *Matrix Reasoning* measures non-verbal fluid reasoning and general intellectual ability. Scores are reported as a Verbal Comprehension Index (VCI), a Perceptual Reasoning Index (PRI), and a Full Scale Intelligence Quotient (FSIQ), which represents performance on all 4 subtests.

The three NPC patients were within the *mild to moderately impaired* range and *moderately to severely impaired* range (Patient 1: FSIQ = 76, VCI = 82, PRI = 74; Patient 2: FSIQ = 62, VCI = 69, PRI = 58; Patient 3: FSIQ = 63, VCI = 72, PRI = 56). Scores on each subtest of the WASI-II are detailed in Table 1. The two older patients exhibited mild high-frequency hearing loss and one of the older patients as well as the younger one had lower than average visual acuity. It is important to emphasize that both auditory and visual stimuli used in the experiment were well above their detectability thresholds. The reader is referred to Table 2 for more comprehensive phenotypic descriptions of each of the three NPC participants.

**Table 1:** **Wechsler Abbreviated Scale of Intelligence scores**

**Wechsler Abbreviated Scale of Intelligence (WASI-II)**	**NPC Participant 1**	**NPC Participant 2**	**NPC Participant 3**
**FULL SCALE IQ (FSIQ)**	**76 (5%)**	**62 (1%)**	**63 (1%)**
**Verbal Comprehension Index (VCI)**	**82 (12%)**	**69 (2%)**	**72 (3%)**
Vocabulary	29	27	31
Similarities	49	34	34
**Perceptual Reasoning Index (PRI)**	**74 (4%)**	**58 (0.3%)**	**56 (0.2%)**
Block design	32	26	28
Matrix reasoning	36	25	21

IQs are standard scores, with a range of 50-160, mean = 100, SD = 15. Corresponding percentile ranks are in parenthesis. Subtests scores (Block Design, Vocabulary, Matrix Reasoning, and Similarities) are T-scores, with a range of 20-80, mean = 50, and SD = 10.

**Table 2:** **Clinical impressions**

**NPC Participant 1**	Participant 1 is a 14 year 8 month old adolescent boy, who was evaluated 3 months after his participation in our behavioral study. He was diagnosed with NPC in 2005 and is currently on the following medications: Zavesca (miglustat), Depakote (divalproex sodium), Keppra (levetiracetam), and Coumadin (warfarin). He has a history of seizures onsetting at age 14. Parental reports indicate clumsiness and unclear speech, which were also observed in the lab. The participant currently receives occupational and speech therapy. He is home-schooled due to the frequency of his seizures. A routine hearing screen performed at the lab revealed mild high frequency hearing loss (i.e. 4,000 Hz tones were not detected at <60 dB & 2,000 Hz tones were not detected at <45 dB). A routine vision screen (Snellen chart) revealed 20/20 and 20/30 visual acuity, in the right and left eyes respectively. Overall intellectual functioning, as measured by the Full Scale IQ on the WASI-II, was estimated in the *mild to moderately impaired* range (FSIQ = 76). His Verbal Comprehension Index score fell in the *mildly impaired* range (VCI = 82) and was somewhat higher than his Perceptual Reasoning Index score which fell in the *mild to moderately impaired* range (PRI = 74); however this difference was not statistically significant. The examiner noted that on several trials of the *Block Design* subtests of the PRI, the participant was able to reproduce the modeled design, however with a 90° rotation. The examiner noted that the participant performed much better when verbal items called for short succinct answers. This likely contributed to his higher *Similarities* score, as several of the relationships probed by the subtest can be addressed with one word explanations, as compared to the *Vocabulary* subtest which requires a more lengthy, developed explanation. Further, the examiner notes that speech was effortful and may have affected performance, with the current scores underestimating the participant’s true abilities. The examiner also noted that the participant appeared fatigued and yawned frequently towards the end of the testing session.
**NPC Participant 2**	Participant 2 is a 14 year 10 month old adolescent boy, who was evaluated 3 months after his participation in our behavioral study. He was diagnosed with NPC in 2005; this patient has a I1061T and M1142T mutation on exons 21 and 22. He is currently on the following medications: Trileptal (oxcarbazepine) and Zavesca (miglustat). He has a history of seizures with the last seizure occurring 10 months prior to testing. The participant currently receives occupational therapy, speech therapy, and has a 1:1 aide at school. A routine hearing screen performed at the lab revealed mild high frequency hearing loss (i.e. 4,000 Hz tones were not detected at <60 dB). A routine vision screen (Snellen chart) revealed 20/60 visual acuity in both eyes. Overall intellectual functioning, as measured by the Full Scale IQ on the WASI-II, was estimated in the *moderately impaired* range (FSIQ = 62). His Verbal Comprehension Index score was in the *mild to moderately impaired* range (VCI = 69) and somewhat higher than his Perceptual Reasoning Index score which fell in the *moderately to severely impaired* range (PRI = 58); however, this difference was not statistically significant. The examiner observed that the participant had motor difficulties when manipulating the blocks used in one of the PRI subtests (*Block Design*). Poor articulation was noted at times, but this was not believed to have interfered with testing.
**NPC Participant 3**	Participant 3 is an 11 year 1 month old boy, who was evaluated on the same day as his participation in our behavioral study. He was diagnosed with NPC in 2013. He is currently on the following medications: Keppra (levetiracetam) and Zavesca (miglustat). He has a history of seizures, including a 4 day hospitalization due to seizure-like activity. He has suffered a concussion that did not render him unconscious. The participant currently receives occupational therapy and academic help with reading and math in a specialized classroom setting at school. Normal hearing was confirmed through a routine hearing screen performed at the lab. A routine vision screen (Snellen chart) revealed 20/50 and 20/30 visual acuity, in the right and left eyes respectively. Overall intellectual functioning, as measured by the Full Scale IQ on the WASI-II, was estimated in the *moderately impaired* range (FSIQ = 63). His Verbal Comprehension Index score fell in the *mild to moderately impaired* range (VCI = 72) and was significantly higher than his Perceptual Reasoning Index score which fell in the *moderately to severely impaired* range (PRI = 56). The examiner noted that the participant had much difficulty with *Block Design* subtest of the PRI, often asking whether the designs presented to him were ‘even possible’. On the *Matrix Reasoning* subtest of the PRI, the participant could not correctly answer any of items at or beyond the starting point for his age and testing here was quickly discontinued. The examiner notes that the participant was pleasant, friendly, and cooperative testing session.

Thirty-five neurotypical boys also participated in this study. Sixteen adolescent boys aged 13-15 served as an age-matched control group for the two older patients. Nineteen boys aged 10-12 served as an age-matched control group for the younger patient. Participants were screened for neurological and psychiatric disorders, as well as other major medical conditions. These data were partially reported in a pair of previous studies [26,34]. Participants were also administered the WASI-II and Full Scale IQ (FSIQ), Verbal Comprehension Index (VCI), and Perceptual Reasoning Index (PRI) scores were obtained, which for these groups were in the *average* or *high average* range (*Older group* mean (standard deviation - SD): FSIQ = 113 (12), VCI = 104 (14), PRI = 110 (12); *Younger group:* FSIQ = 113 (14), VCI = 108 (12), PRI = 113 (13)). Audiometric evaluation confirmed that all participants had within-normal-limits hearing thresholds. All participants had normal or corrected-to-normal vision.

Before entering into the study, informed written consent was obtained from the children's parents, and verbal or written assent was obtained from children. All procedures were approved by the Institutional Review Board at The Albert Einstein College of Medicine and were in accordance with the tenets for the responsible conduct of human research laid out in the Declaration of Helsinki.

### Paradigm & task

#### Stimuli

##### Auditory alone

A 1000-Hz tone (duration 60 ms; 75 dB SPL; rise/fall time 5 ms) was presented from a single Hartman Multimedia JBL Duet speaker located centrally atop the computer monitor from which the visual stimulus was presented.

##### Visual alone

A red disc with a diameter of 3.2 cm (subtending 1.5° in diameter at a viewing distance of 122 cm) appearing on a black background was presented on a Liquid Crystal Display (LCD) monitor (Dell Ultrasharp 1704FTP, 60Hz refresh rate) for 60 ms. The disc was located 0.4 cm superior to central fixation along the vertical meridian (0.9° at a viewing distance of 122 cm). A small cross marked the point of central fixation on the monitor.

##### Auditory and visual simultaneous

The “auditory-alone” and “visual-alone” conditions described above were presented simultaneously. The auditory and visual stimuli were presented in close spatial proximity, with the speaker placed atop the monitor in vertical alignment with the visual stimulus.

### Procedures

Participants were seated in a dimly lit, sound-attenuated electrically shielded room (Industrial Acoustics Company, Bronx, New York) 122 cm from the monitor. They were given a response pad (Logitech Wingman Precision) and instructed to press a button with their right thumb as quickly as possible when they saw the red circle, heard the tone, or saw the circle and heard the tone together. The same response key was used for all 3 stimulus types. Presentation software (Neurobehavioral Systems, Inc., Albany CA) was used for stimulus delivery. This software ensures precise timing of stimulus presentation and is commonly used in neuroscience, psychophysics, and psychological experiments. It takes into account the refresh rate of the computer monitor when presenting visual stimuli. In this experiment, stimulus delivery in the multisensory condition was triggered by the onset of the visual stimulus. All 3 stimulus types were presented with equal probability and in random order in blocks of 100 trials. Inter-stimulus-interval (ISI) varied randomly between 1000 and 3000 (ms) according to a uniform (square wave) distribution. Participants completed a minimum of 8 blocks, with most completing 10. Breaks were encouraged between blocks to help maintain concentration and reduce restlessness or fatigue (these methods are also presented in detail in Brandwein et al [26,34] and Molholm et al [21]).

### Interrogating the race model

To test the race model, we first calculated the cumulative probability of reaction times across the three stimulus types (audio-alone, visual-alone, and audio-visual) for each of the participants. The range of RTs accepted was determined at the individual participant level with the slowest and fastest 2.5% of trials excluded. Using a 95% cutoff to define the time window for acceptable trials rather than an absolute cutoff value allowed us to more accurately capture the range of RTs for each participant, an important factor in calculating the race model (described below). The RT distribution was then divided into quantiles from the 5th to the 100th percentile in increments of 5%. For any RT latency, *t*, the race model holds when this CP value is less than or equal to the sum of the CP from each of the unisensory conditions. Conversely, the race-model is said to be violated if the CP for any audiovisual RT latency is larger than that predicted by the race model (the sum of the unisensory CPs) at any quantile. Violations were expected to occur in the first third of the distribution (i.e. the quantiles containing the fastest RTs at the lower end of the RT range) because this is when interactions between visual and auditory inputs would result in the fulfillment of a response criterion before either input alone could satisfy the same criterion [31]. At the individual level, a participant was said to have shown race model violation if the CP of his RT to the audiovisual stimulus was larger than that predicted by the race model at any quantile within the first third of the distribution. In order to more easily interpret results from the race model test, a Miller inequality value can be computed, both at the individual and group levels, by subtracting the CP predicted by the race model from the CP of the multisensory condition. Any positive “Miller values” indicate race model violation and RT speeding that cannot be accounted for by probability summation or by the *‘redundant signals effect’*.

## Results

### Behavioral performance - reaction times & hit rates

**Figure 1:**
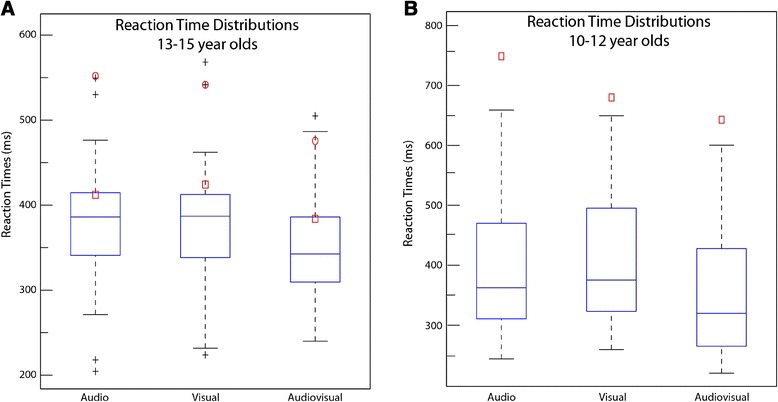
**Reaction time box and whisker plots.** The plots show the distributions of mean RT values for 13-15 year olds (Panel **A**) and for 10-12 year olds (Panel **B**), for the two unisensory (Audio and Visual) and the multisensory (Audiovisual) conditions. The red symbols represent the mean RT values for each of the Niemann-Pick type C participants and the black crosses represent mean RT values for individual outliers from the neurotypical groups.

The neurotypical group had a higher percentage of hits (correctly pressing the button to stimulus presentations) than the NPC participants. Hit rates are presented in Table 3. The current report was primarily concerned with the speed of responding. Overall, neurotypical participants were faster than the NPC patients (Table 4 and Figure 1). In order to examine RT variability independent of mean RT differences between the groups and between experimental conditions, the coefficient of variation (CV) was calculated for auditory, visual, and audiovisual conditions for each individual participant. The CV for the older patients fell within the neurotypical distribution or overlapped with individual neurotypical outliers. The CV for the younger patient fell outside (but close) to the neurotypical distribution; however there were also younger neurotypical controls that were more variable than this younger patient (see Additional file 1). What's more, in both neurotypical age-groups, variability was greatest for the auditory condition and did not differ significantly between the two other conditions. Observationally, the CV for individual NPC patients did not appear to differ substantially across experimental conditions. Nonparametric tests revealed no significant differences in RT variability based on stimulus type. Thus, increased variability in the multisensory condition should not affect the race model analysis presented below (for a Discussion see [37]). Detailed analyses and figures related to CV are provided in Additional file 1.

**Table 3:** **Hit rates**

	**Auditory**	**Visual**	**Audio-visual**
NPC Participant 1	59%	60%	62%
NPC Participant 2	78%	73%	83%
NPC Participant 3	57%	63%	68%
**Older neurotypicals (13-15 years old; N = 16)**	92% (3)	91% (4)*	93% (2)*
**Younger neurotypicals (10-12 years old; N = 19)**	91% (4)*	88% (6)*	91% (4)*

*Hit rates are depicted as a percentage reflecting correct responses divided by total number of stimuli presented, with the standard deviations in parenthesis for the neurotypical group data. For the NPC participants hit rates is a within subject value and therefore has no SD.

**Table 4:** **Reaction times**

	**Auditory**	**Visual**	**Audio-visual**
NPC Participant 1	416 (218)	426 (156)	387 (168)
NPC Participant 2	555 (282)	545 (277)	472 (225)
NPC Participant 3	749 (440)	680 (374)	643 (397)
**Older neurotypicals (13-15 years old; N = 16)**	379 (95)*	381 (93)*	348 (79)*
**Younger neurotypicals (10-12 years old; N = 19)**	390 (109)*	404 (109)*	341 (102)*

*Reaction times are given in milliseconds with the standard deviations in parenthesis. For the NPC participants the SD reflect a within subject value. For the neurotypicals the SD is computed on the group mean.

A repeated measures ANOVA revealed a significant effect of stimulus type on RTs for both the older F(2,30) = 12.1, *p* < .001 and younger F(2,36) = 91.4, *p* < .001 neurotypical groups. Follow-up protected t-tests confirm a speeding up of RTs for the multisensory condition for the older neurotypical group (*Audio vs. AV* - t(15) = 3.4, *p* < .01; *Visual vs. AV* - t(15) = 5.0, *p* <. 01; *Audio vs. Visual* - t(15) = -.31, *p* = .76) and for the younger neurotypical group (*Audio vs. AV* - t(18) = 10.4, *p* < .01, *Visual vs. AV*- t(18) = 12.4, *p* < .01). Additionally, the younger group had significantly faster RTs to the auditory condition as compared to the visual condition, t(18) = -3.1, *p* < .01.

**Figure 2:**
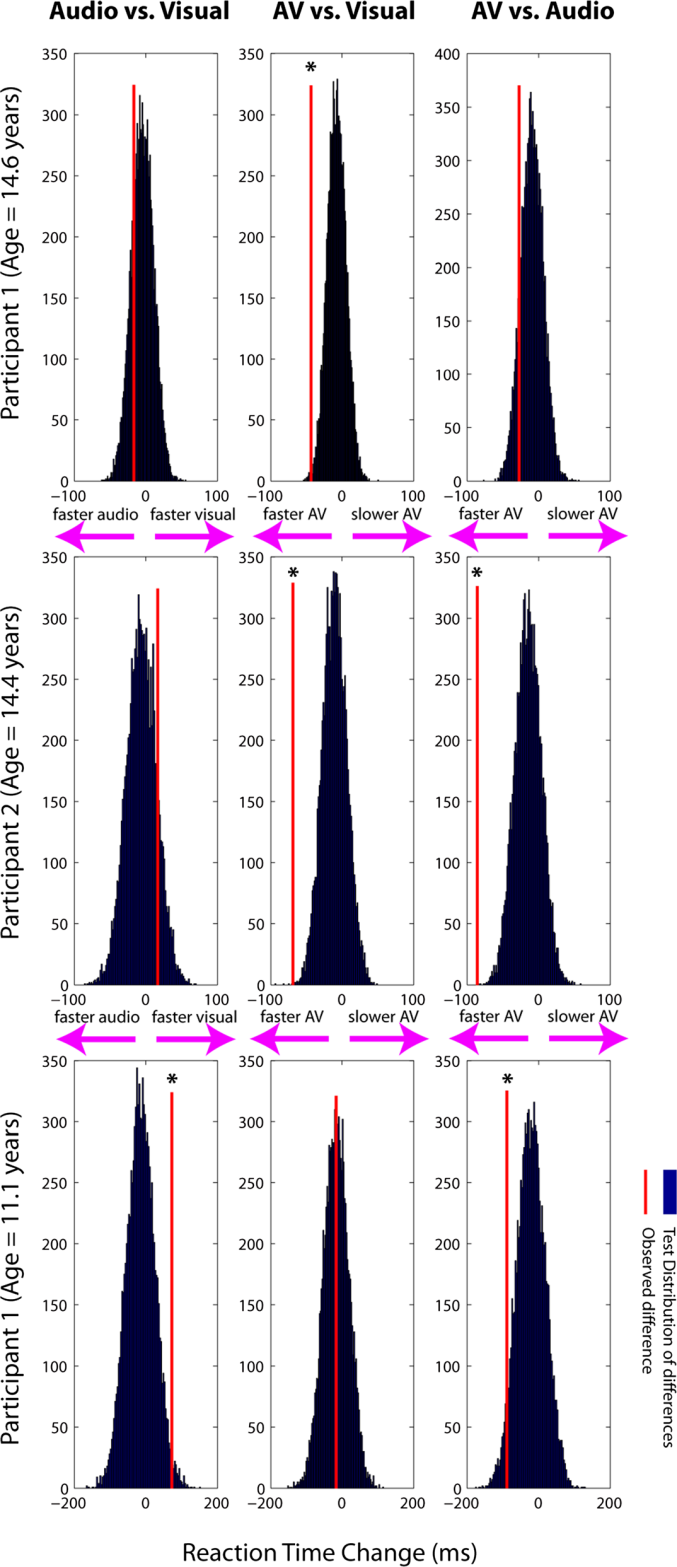
**Nonparametric randomization plots for the individual-participant reaction time data for each Niemann-Pick type C patient.** RTs in each of the unisensory conditions were compared against the multisensory RTs (middle and right columns), and against each other (left column). The observed differences in mean RT between *Audio vs. AV*, *Visual vs. AV, and Audio vs. Visual* (red line) were compared with reference distributions of differences that were derived by iteratively randomizing (10,000 times) between the two original data sets (i.e. individual-subject single trial RTs for 1) Audio and AV, 2) Visual and AV, and 3) Audio and Visual)*.* Significant differences (*p* < .05) are indicated by an asterisk. The findings are mixed. In two of the three patients, any apparent multisensory speeding is not significantly faster than the faster of the two unisensory responses. However, in one of the patients (Participant 2), RTs to the AV condition are significantly faster compared to both unisensory inputs. This particular patient is showing strong evidence for the so-called redundant sensory effect, but this speeding does not violate the race model.

As our NPC sample contained only 3 participants, we performed a nonparametric bootstrapping procedure at the level of the individual participant data to compare RTs across the three sensory conditions (Figure 2). For each NPC patient, we compared the RTs in each of the unisensory conditions against the multisensory RTs, as well as against each other. The observed differences in mean RT between *Audio vs. AV*, *Visual vs. AV,* and *Audio vs. Visual* were compared with reference distributions of differences that were derived by iteratively randomizing (10,000 times) between the two original RT distributions - i.e. individual-subject single trial RTs for 1) Audio and AV, 2) Visual and AV, and 3) Audio and Visual. A two-tailed threshold of *p* <0.05 was used to define significance. The *p* value for a randomization test was calculated from the proportion of values in the reference difference distribution that exceeded the actual observed difference. In other words, we created a randomized sample distribution of possible reaction time differences, and sought to determine the likelihood that the actually observed differences (either speeding up or slowing down) were due to chance. There was no significant difference between auditory and visual RTs for the older NPC participants. The younger participant (Participant 3) showed significantly faster RTs in the visual condition compared to the auditory (*p* = .015). A significant speeding up was noted in the multisensory condition relative to the visual condition (*p* < .01), but not the auditory condition, for Participant 1. This was likely driven by the response to the auditory stimulus as the speeding up is only significant in the AV vs. V comparison. A significant speeding up was noted in the multisensory condition relative to the auditory condition (*p* < .05), but not the visual condition, for Participant 3. Again, this was likely driven by the response to the visual stimulus as the speeding up is only significant in the AV vs. A comparison. A significant speeding up in the multisensory condition compared to both unisensory conditions (*p's* < .01) was noted for Participant 2, indicating the presence of a *Redundant Signals Effect*. These tests, however, do not take into account facilitation due to multisensory interactions, which will be tested below using the race model calculation.

If motor difficulties alone were to account for the larger variance in RTs and lower hit rates in the NPC participants, one would expect these to occur at the same probability across all three experimental conditions, which is not the case in this sample. Deficits in motor response do not account for the differential effect noted in 2 of the patients across the unisensory and multisensory conditions. The two NPC adolescents had faster RTs and a higher percentage of hits in the multisensory conditions compared to the unisensory. To probe the nature of this speeding up and assess whether the patients may be benefitting from an integrative process, we applied a test for multisensory integration effects (i.e. testing the race model). In this test a within-individual analysis is employed, thus accommodating the between group differences already noted.

### Multisensory integration effects - race model

**Figure 3:**
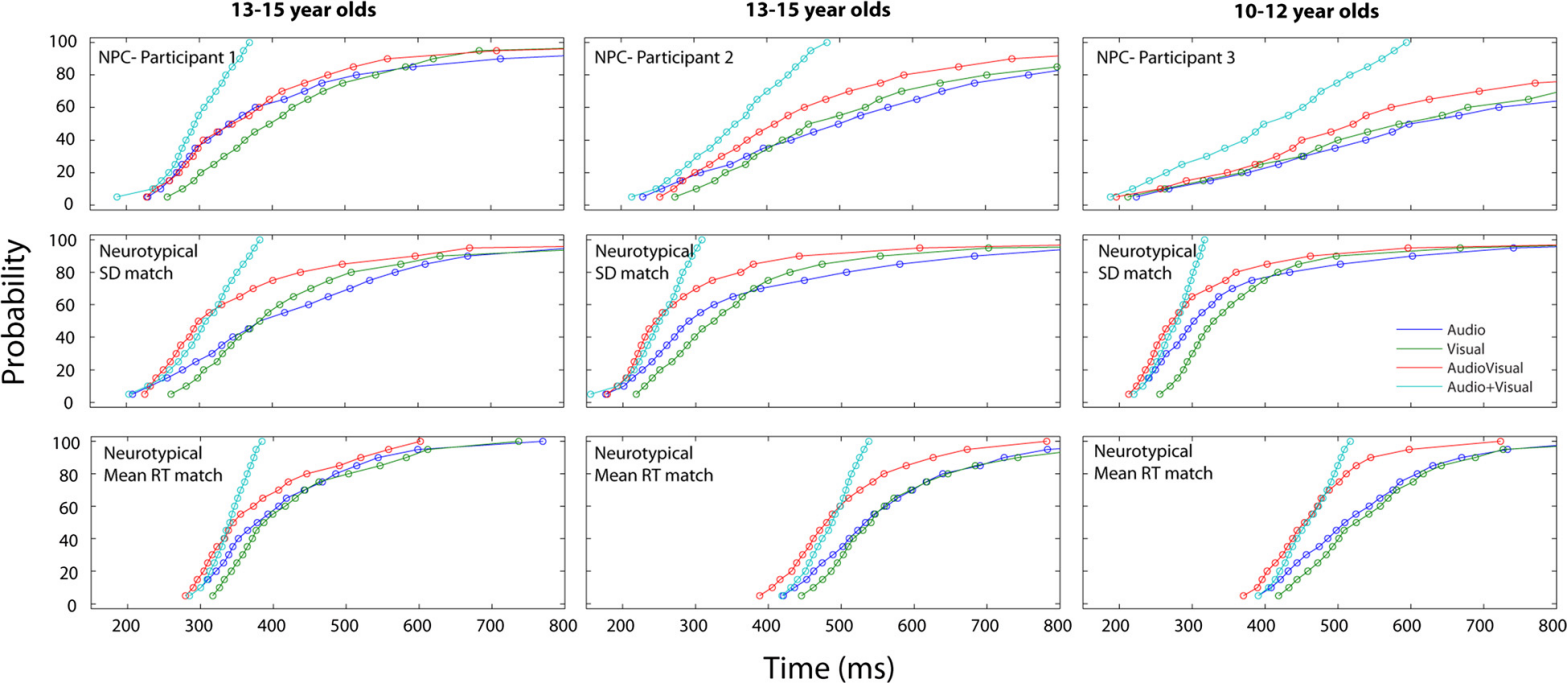
**Cumulative reaction time (RT) probability distributions.** The cumulative probability of RTs for the three Niemann-Pick type C patients (top row) are compared to those of six neurotypical boys. The three age-matched comparison subjects depicted along the middle are chosen for their highly similar RT variance. The bottom row depicts three age-matched controls chosen for their highly similar mean RTs to those of the NPC boys. In the case of all six neurotypical controls, the observed cumulative RT distribution to the multisensory audio-visual condition (red curve) is faster than the prediction of the race model (cyan curve), indicating race model violation (i.e. multisensory integration). In none of the three NPC cases is this pattern observed.

None of the three NPC participants showed any evidence of race model violation. Although in some cases, they showed faster RTs in the audiovisual condition (see above), this was not greater than could be accounted for by simple probability summation. In stark contrast, all of the neurotypical adolescents in our older sample of 13-15 year olds showed individual-level race model violation, suggesting that in this age group, multisensory integration reliably improves behavioral performance under these conditions. For the 11 year old NPC patient, an additional cutoff criterion was applied to his RT data before computing the race model. Unlike the rest of our sample, even after excluding the fastest 2.5% of RTs, this participant still had several anticipatory RTs that would be physiologically impossible (i.e. response latencies in the 40-100 ms range). These anticipatory responses were evenly distributed across all stimulus conditions (12% of the Audio trials, 13.5% Visual trials, and 10% of the AV trials). In order to eliminate any button presses that weren't directly in response to the stimulus, a hard cutoff criteria of 150ms was employed in his case, as it is generally agreed upon that shorter response latencies indicate actions that were initiated before the stimulus onset [38–44]. In the younger sample of 10-12 year olds, 16 of the 19 participants showed individual-level race model violation. Figure 3 depicts the CP distributions of reaction times for each of the experimental conditions -- audio-alone (blue), visual-alone (green), audiovisual (red), and the race model prediction (using the sum of the CPs of the unisensory responses (teal). Data for the three NPC boys are depicted across the top row. Across the middle row, data from three neurotypical individuals whose RT variability closely matched that of the NPC children are plotted for comparison. Despite similar RT variance, each of these neurotypicals shows race model violation. The bottom row shows data from an additional three neurotypical boys, where RT mean has been matched to each of the NPC boys. Again, all 3 neurotypicals show clear race model violation.

**Figure 4:**
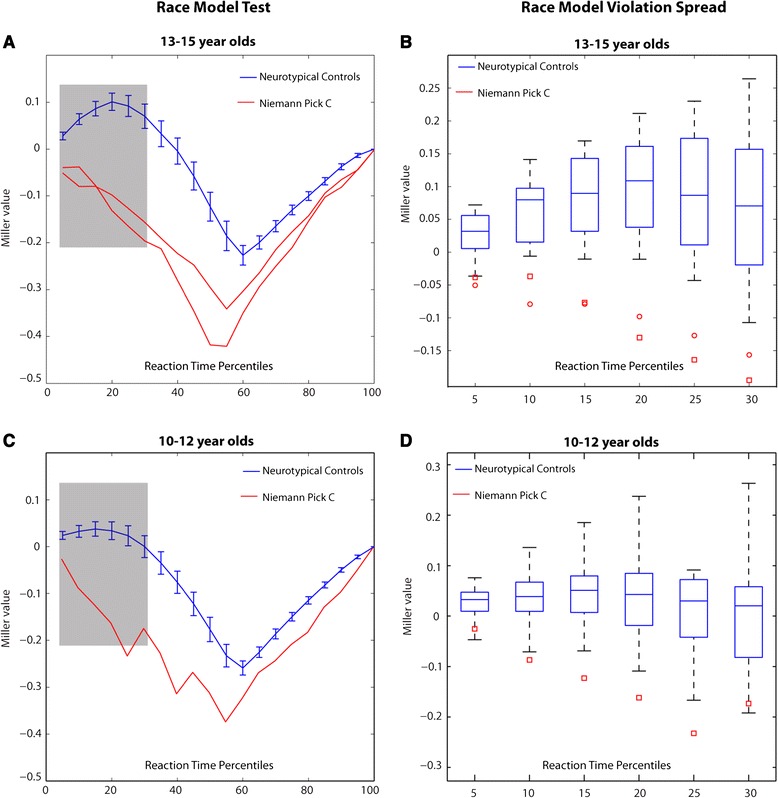
**Race model test and Miller value spread. A & C)**. Race model plots depict the Miller value for the neurotypical groups (blue curves) and the Niemann-Pick type C patients (red curves). Values above zero indicate race model violation, which are evident in both the older neurotypicals (N = 16; Panel A) and the younger neurotypicals (N = 19; Panel B), but not in the NPC patients. The shape of the Miller inequality plot observed in the NPC patients is highly atypical and consistent across all three patients. **B & D)**. Box and Whisker plots depict the spread of Miller values for the first 6 RT quantiles for the neurotypical group and the single subject Miller values for each of the NPC adolescents (red circles and red squares). This plot depicts the spread of Miller values for approximately 99% of both neurotypical groups, with the box representing 50% of the data, the whiskers representing the top 25 and bottom 25 percent, and the horizontal bisecting line representing the median Miller value for each neurotypical group at that quantile. It can be seen that all three NPC patients (red shapes) fall outside of the distribution of Miller values for their age-matched neurotypical group. Multisensory facilitation at the individual participant level was noted in all 16 of the 13-15 year olds and in 16 of 19 of the 11-12 year old neurotypicals.

Figure 4A & 4C depict plots of “Miller inequality” values which were obtained by subtracting the CP predicted by the race model from the CP for the multisensory condition. Positive values represent race model violation. Here it can be seen that the traces representing the two older NPC participants (4A- red) are never positive, whereas the trace representing the older neurotypical controls (blue) is positive for the quantiles representing the fastest ~30% of RTs. The shape of this Miller inequality function for neurotypical controls is highly similar to those reported in similar studies examining audio-visual integration [26,34]. The Miller inequality plot for the younger neurotypical controls (Figure 4C- blue) closely approximates the pattern seen in the older children, albeit more immature. In the younger NPC participant, no race model violation is noted and the shape of his Miller inequality plot has the same atypical pattern noted in the two older NPC participants. Figure 4B & 4D depict box and whisker plots, which offer an additional representation of these data. Here the box and whiskers (blue rectangles with black bars) represent the Miller inequality values for all of the participants in the neurotypical group for the first six quantiles, which is the section of the RT distribution containing the fastest responses and also where race model violations are expected and seen in the neurotypical group (shaded area in Figure 4A & 4C). The small red shapes (squares and circles) represent the Miller inequality values for each NPC participant at these quantiles. This plot clearly shows that all three NPC boys fall completely outside the normal distribution between the second and sixth quantiles (10th, 15th, 20th, 25th, and 30th percentiles). Although race model violation is seen from the first quantile onward for the neurotypical participants, it is not necessarily seen for all participants at the exact same quantiles. That is to say that some participants will show race violation sooner than others and some will continue to show race model violation for several quantiles while the effect for others will dissipate more quickly. These effects, however, are generally seen in first third of the CP distribution as interactions between auditory and visual stimuli are likely to occur during these shorter latencies and so here we focus on the first 5 quantiles of this distribution. Further, we note that multisensory facilitation, as evidenced by race model violation (i.e. Miller inequality value greater than 0) was noted at the individual participants level for all 16 of the 13-15 year old neurotypical controls. For the 10-12 year olds, an age in which multisensory integration is still emerging and somewhat immature [45,46], individual-level race model violation was seen for 16 out of 19 (84%) neurotypical controls. The NPC participants, on the other hand, failed to violate the race model at any point along the CP distribution. This lack of race model violation is especially striking for the older NPC participants as mean RT values for these NPC participants fall well within the neurotypical distribution in the case of one of the NPC patients, and overlaps with neurotypical outliers for the other patient (Figure 1A). This suggests a true multisensory deficit in that AV gains are accounted for by probability summation *and* there are no clear overall unisensory deficits contributing to this finding. For the younger participant, this is harder to say as his mean RTs for the auditory and the AV conditions fall slightly outside the neurotypical distribution. Nonetheless, the gains noted in his case can be adequately explained without evoking multisensory interactions as they are no greater than that predicted by probability summation.

## Discussion

To our knowledge, this is the first study to examine multisensory processes in NPC. The observed lack of race model violation in NPC suggests compromised connectivity between auditory and visual areas of the brain, possibly at both sub-cortical and cortical levels. It is likely that these inter-sensory connections develop very early in life, strengthen across childhood, and stabilize during adolescence [26,34,47,48]. Understanding when exactly during the progression of NPC that MSI becomes compromised will require further investigation and will be crucial to maximizing the clinical usefulness of this measure in the NPC population. Two possible scenarios are that; 1) MSI-induced behavioral facilitation never quite reaches “healthy” levels in these individuals or 2) that like many of the other symptoms exhibited in this population, NPC patients experience a degradation of MSI function with progression of the disease state. In either case, this metric of MSI presents a behavioral marker against which to measure improved neurocognitive function due to experimental treatment interventions.

In terms of everyday functioning, an obvious question is what impact deficits in multisensory processing will have on the abilities of NPC children to effectively navigate their environment. For example, effective MSI leads to improved speech perception when a listener has the benefit of watching the facial articulations of a speaker, especially if the fidelity of the auditory input is affected by noisy background environmental conditions [17,22,23,49,50]. Thus, one implication is that these children may find communication more difficult in challenging multi-speaker scenarios, not uncommon in classrooms or other social settings. MSI is also vital to more basic functions, such as maintaining balance through visuo-vestibular and visual-somatosensory integration [15] and in speeded orienting to reliable multisensory events, whether it be for object identification or cueing initiation of approach/avoidance behaviors [16,18–21,24]. A more comprehensive understanding of the multisensory integration abilities of these children is clearly called for, and it will be of significant interest to assess the underlying neurophysiology in turn [51,52].

Another obvious outcome of the current study is that the NPC children show basic motor deficits. While it is true that there are neurotypical participants who are as slow to respond to unisensory inputs, and others who show similarly high variance in RTs, no neurotypical children show the poor response rates we see in the NPC children. Simply put, the NPC children are slow, variable and inaccurate and this triumvirate of issues clearly points to fundamental sensory-motor issues. That said, we do not believe that the MSI deficits observed here are primarily due to these issues, since these issues apply equally to all the experimental conditions (both unisensory and multisensory; also see Additional file 1). As the race model analysis is conducted at the individual participant level, where the cumulative probability distributions are calculated for each participant and within-subject RTs are compared to determine the multisensory benefit, general motor delays are accounted for. It could reasonably be asked, though, whether simple tests of motor speed, variance and accuracy might not prove equally useful biomarkers for NPC. However, it bears re-emphasizing that while the NPC children do show these issues, their performance levels do not fall completely outside the normal distribution for these measures, whereas for the measures of multisensory integration, they clearly do.

It is worth pointing out that these children with NPC are, at some basic level, benefitting from multisensory stimulation, even if not in an integrative manner. The fact that mean RTs and hits are improved in some cases, even in the absence of significant multisensory integration, when patients are exposed to stimulation in two sensory streams is promising, especially in terms of sensory training. This may have implications for the development of assistive technologies used for communication, particularly during the more progressed phases of the disease.

A natural question that arises is whether the multisensory deficit we observe in NPC can be meaningfully impacted through intervention. The landscape is actually quite promising in this regard since several studies now point to multisensory and unisensory gain with repeated training. These studies show that training can lead to improvement in MSI-dependent tasks such as speech-perception [53], that training can narrow the time window during which two sensory inputs are seen as “synchronous” and thus integrated [54], and that MSI networks can be engaged and enhanced in training activities where abstract stimuli are paired, such as specific sounds with abstract shapes, or musical tones with symbols [55,56]. Work in animal models also supports the notion that sensory integration abilities can be impacted through practice with training-induced multisensory enhancement noted in both behavior and activity patterns at the single cell level in the superior colliculus, in both juvenile [57] and adult cats [58].

An obvious limitation of the current work is the relatively small cohort of three patients with NPC that we were able to test. Ideally, one would like to have greater numbers. However, the disease prevalence rate for NPC is estimated at 1-in-120,000 [6,8,59], so recruitment of larger populations is extremely challenging. It is worth emphasizing that the atypical multisensory integration pattern noted here is highly consistent across the 3 NPC patients in our sample and the findings are strengthened by comparison of these 3 patients to large existing datasets of neurotypical age-matched children. In all 3 cases, the performance metrics of the NPC patients fall completely outside the “normative” curve for MSI development.

## Conclusions

This study uncovered clear multisensory deficits in three patients with NPC. The simple-to-acquire measures of multisensory response speed described here may prove to be useful endpoints against which to track disease progression and to assess the efficacy of therapeutic interventions. Specific environmental accommodations should be considered to address the potential impact of deteriorating multisensory mechanisms in these children.

## Additional file

Additional file 1:
**Variability analysis. Figure S1.** Coefficient of Variation Spread. **Table S1.** Coefficient of Variation.
