# Supplementary material for: Atypical multisensory integration in Niemann-Pick type C disease – towards potential biomarkers
Source: Orphanet J Rare Dis. 2014 Sep 20;9:149. doi: 10.1186/s13023-014-0149-x (PMC4173006; doi:10.1186/s13023-014-0149-x)
Supplement: Additional file 1: — Variability analysis. Figure S1. Coefficient of Variation Spread. Table S1. Coefficient of Variation. [file 13023_2014_149_MOESM1_ESM.docx]

**Additional file**

***Variability Analysis***

*We calculated the coefficient of variation (CV) for RTs for each individual in each of the three sensory conditions. Mean and standard deviation of the CV are presented for both neurotypical groups below, along with the individual values for the three NPC patients. We also provide a boxplot figure depicting the spread of the CV for the neurotypical groups along with the CV values for the individual NPC patients. We see that the CV for the older patients either falls within the neurotypical distribution or overlaps with individual neurotypical outliers. The CV for the younger patient falls outside (but close) to the neurotypical distribution; however there were also younger neurotypical controls that were more variable than this patient.*

*Interestingly, the plots also suggest that, RT variability was smallest in the multisensory condition. For the neurotypical groups we formally tested this using a repeated measures ANOVA which revealed a significant effect of stimulus type on CV for the older (F (2, 30) = 9.71, p = .001) and younger (F(2, 36) = 19.58, p < .001) neurotypical groups. Follow-up protected t-tests show significantly higher variability, as measured by the coefficient of variation, for the auditory condition only for the older neurotypical group (Audio vs. Visual - t(15)= 3.7, p = .002; Audio vs. AV - t(15)= 3.46, p =. 004; Visual vs. AV - t(15)= 1.2, p= .24) and as well as the younger neurotypical group (Audio vs. Visual - t(18)= 4.41, p < .001, Audio vs. AV- t(18)=5.28, p < .001, Visual vs. AV - t(18)= .74, p= .47). To reiterate, we didn't observe any evidence of "increased noise" in the multisensory RTs.*

*As our NPC sample contained only 3 participants, we are unable to conduct the same set of analyses for the patients. However, visual inspection of the coefficient of variation spread plots show that the older NPC boys seem to follow the same pattern observed in the neurotypical controls. Both of the older boys show the highest variation in RTs in the auditory condition, as with no obvious increase in CV in the multisensory condition. The younger NPC participant does not appear to follow this trend. To better understand CV across the three sensory conditions in this small sample we employed a nonparametric test. A Related-Samples Friedman ANOVA by ranks test on the NPC patient data (treating them as a single group, N=3) showed no significant difference in the coefficient of variation between the three experimental conditions (p .44). This is a reassuring finding and seemingly in line with the individual patient data, however this is an N of 3 sample and clearly more work needs to be done before the relationship between 'noise,' variability, and multisensory facilitation is understood in this population. For a discussion on the role of noise in MSI see (*[*1-3*](#_ENREF_1)*).*

*Figure Captions*

***Figure S1.***

*Box and whisker plots show the distributions of the coefficient of variation values for 13-15 year olds (Panel A) and for 10-12 year olds (Panel B), for the two unisensory (Audio and Visual) and the multisensory (Audiovisual) conditions. The red symbols* *represent the CV values for each of the Niemann-Pick Type C participants and the blue crosses represent mean RT values for individual outliers from the neurotypical groups.*

**Figure S1.** Coefficient of Variation Spread


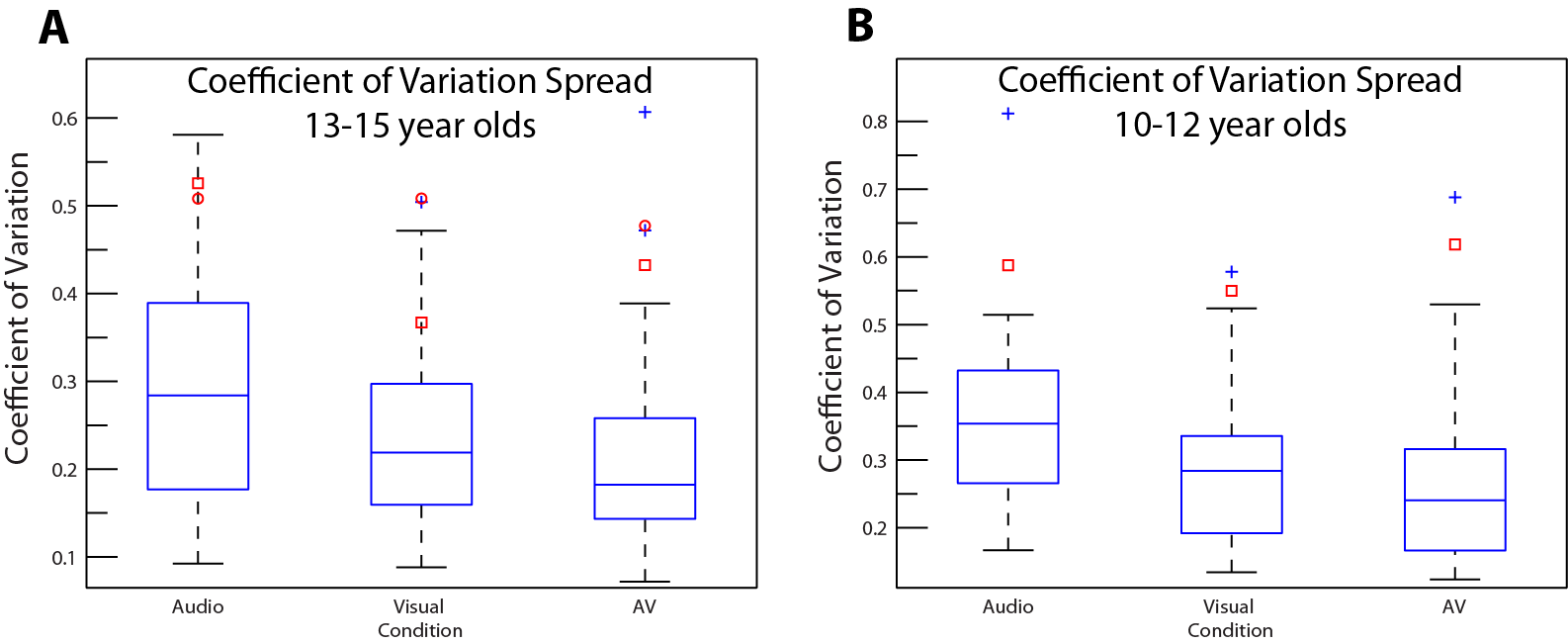


**Table S1.** Coefficient of Variation

|  | *Auditory* | *Visual* | *Audio-visual* |
| --- | --- | --- | --- |
| NPC Participant 1 | .53 | .37 | .43 |
| NPC Participant 2 | .51 | .51 | .48 |
| NPC Participant 3 | .59 | .55 | .62 |
| **Older Neurotypicals (13-15 years old; N=16)** | .29 (.14) | .24 (.12) | .23 (.15) |
| **Younger Neurotypicals**  **(10-12 years old; N=19)** | .36 (.15) | .29 (.19) | .28 (.14) |

**REFERENCES**

1. Barutchu, A., Crewther, D. P. & Crewther, S. G. (2009) The race that precedes coactivation: development of multisensory facilitation in children, *Developmental Science*, 12, 464-473.

2. Otto, T. U. & Mamassian, P. (2012) Noise and correlations in parallel perceptual decision making, *Curr Biol*, 22, 1391-6.

3. Senkowski, D., Saint-Amour, D., Hofle, M. & Foxe, J. J. (2011) Multisensory interactions in early evoked brain activity follow the principle of inverse effectiveness, *Neuroimage*, 56, 2200-8.
